# Magnetic microgels and nanogels: Physical mechanisms and biomedical applications

**DOI:** 10.1002/btm2.10190

**Published:** 2020-10-21

**Authors:** Baeckkyoung Sung, Min‐Ho Kim, Leon Abelmann

**Affiliations:** ^1^ KIST Europe Forschungsgesellschaft mbH Saarbrücken Germany; ^2^ Department of Biological Sciences Kent State University Kent Ohio USA; ^3^ Division of Energy and Environment Technology University of Science and Technology Daejeon Republic of Korea; ^4^ MESA+ Institute for Nanotechnology, University of Twente Enschede The Netherlands

**Keywords:** functional nanoparticle, magnetic field, polymer network, smart hydrogel

## Abstract

Soft micro‐ and nanostructures have been extensively developed for biomedical applications. The main focus has been on multifunctional composite materials that combine the advantages of hydrogels and colloidal particles. Magnetic microgels and nanogels can be realized by hybridizing stimuli‐sensitive gels and magnetic nanoparticles. They are of particular interest since they can be controlled in a wide range of biological environments by using magnetic fields. In this review, we elucidate physical principles underlying the design of magnetic microgels and nanogels for biomedical applications. Particularly, this article provides a comprehensive and conceptual overview on the correlative structural design and physical functionality of the magnetic gel systems under the concept of colloidal biodevices. To this end, we begin with an overview of physicochemical mechanisms related to stimuli‐responsive hydrogels and transport phenomena and summarize the magnetic properties of inorganic nanoparticles. On the basis of the engineering principles, we categorize and summarize recent advances in magnetic hybrid microgels and nanogels, with emphasis on the biomedical applications of these materials. Potential applications of these hybrid microgels and nanogels in anticancer treatment, protein therapeutics, gene therapy, bioseparation, biocatalysis, and regenerative medicine are highlighted. Finally, current challenges and future opportunities in the design of smart colloidal biodevices are discussed.

**Abbreviations**
BMP‐2: bone morphogenetic protein 2
LCST: lower critical solution temperature
MG: microgel
miRNA: microRNA
MMG: magnetic microgel
MNG: magnetic nanogel
MNP: magnetic nanoparticle
MPI: magnetic particle imaging
MRI: magnetic resonance imaging
NG: nanogel
NIPAM: *N*‐isopropyl acrylamide
NP: nanoparticle
PEG: poly(ethylene glycol)
PNIPAM: poly(*N*‐isopropyl acrylamide)
siRNA: small interfering RNA
UCST: upper critical solution temperature

## INTRODUCTION

**FIGURE 1:**
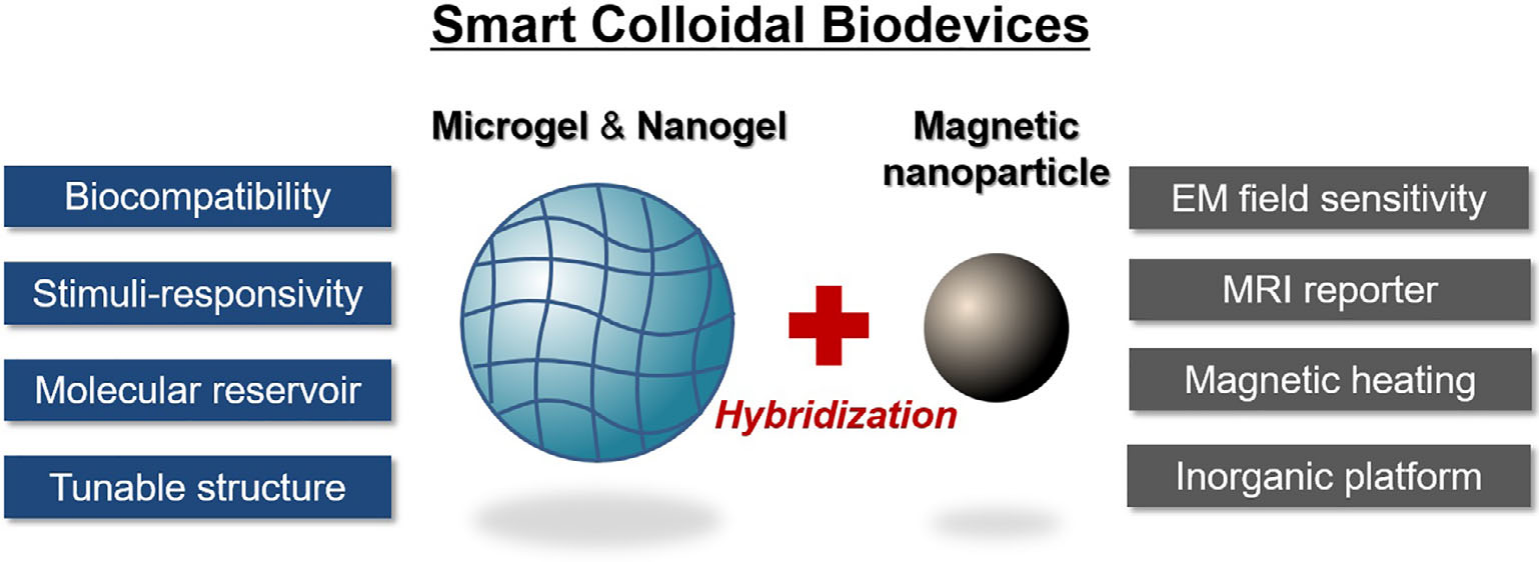
Hybrids of microgels/nanogels and magnetic nanoparticles as smart colloidal biodevices

Over the past two decades, stimuli‐responsive polymers and multifunctional nanoparticles have been increasingly used for the fabrication of nanometer‐ and micrometer‐scale soft devices for a range of biomedical applications and clinical fields.^1^, ^2^, ^3^, ^4^, ^5^ In particular, composites of polymeric micro‐ and nanohydrogels with magnetic nanoparticles (MNPs) have attracted increasing attention in the fields of nanomedicine and tissue engineering. These composites are highly sensitive and show rapid reactivity to local multicellular environments and external stimuli, such as temperature, pH, and electromagnetic fields, and they are promising candidates for programmable and remotely controllable (and trackable) biomaterial components that can be introduced in a living body or in culture cells and tissues (Figure 1).

Despite the rapid expansion of the field of miniaturized hydrogels and nanobiomaterials and increasing demand for commercialization from pharmaceutical industries, our current understanding on the physical mechanisms behind each specific application is limited.^6^, ^7^ To remedy this situation and provide further breakthroughs, it is essential to bridge the subjects of microgels (MGs) and nanogels (NGs) to functional MNPs. Furthermore, grasping how the relationships between miniaturized gels and MNPs are applied in the field of biomedicine is critical for employing hybridized magnetic MGs and NGs (hereafter abbreviated as MMG and MNG, respectively) for specific biomedical purposes in vivo and in vitro. The development of future soft biodevices should be based on the designing and modeling, similar to those of solid‐state electronic devices on flexible and stretchable platforms.^8^ This review article attempts to contribute to the realization of this vision by systematically describing the physical engineering principles of MMG and MNG systems underlying the design of magnetic soft gel device. Particularly, this article provides a comprehensive and conceptual overview on the correlative structural design and physical functionality of the systems in the context of translational biomedical applications, which has not been well addressed in the literature.

To this end, we focus on the major physicochemical mechanisms of these hybrid systems in Part I, and discuss recent progress in biomedical applications of the hybrids of MG/NG and MNPs. Recent developments in controlled drug release, cancer therapeutics, protein and gene delivery, and bioseparation and biocatalysis are discussed in close relation to the engineering principles introduced in Part I.

## PART I: PHYSICAL MECHANISMS AND ENGINEERING PRINCIPLES

In the following, we first introduce the coil‐globule transition of polymers, which occurs in most responsive hydrogels, and then describe the basic thermodynamic concepts, phase transition, and swelling kinetics of polymeric hydrogels. This information provides the fundamental basis for hydrogel responsivity to microenvironmental stimuli. Elementary concepts pertaining to diffusion phenomena in hydrogels are additionally described in relation to controlled drug delivery through static or dynamic drug release from hydrogels. In the next section, we discuss the magnetism of inorganic MNPs, with emphasis on their biomedical applications. Finally, we discuss the hybridization of MGs and NGs with MNPs.

### Stimuli‐responsive hydrogels

**FIGURE 2:**
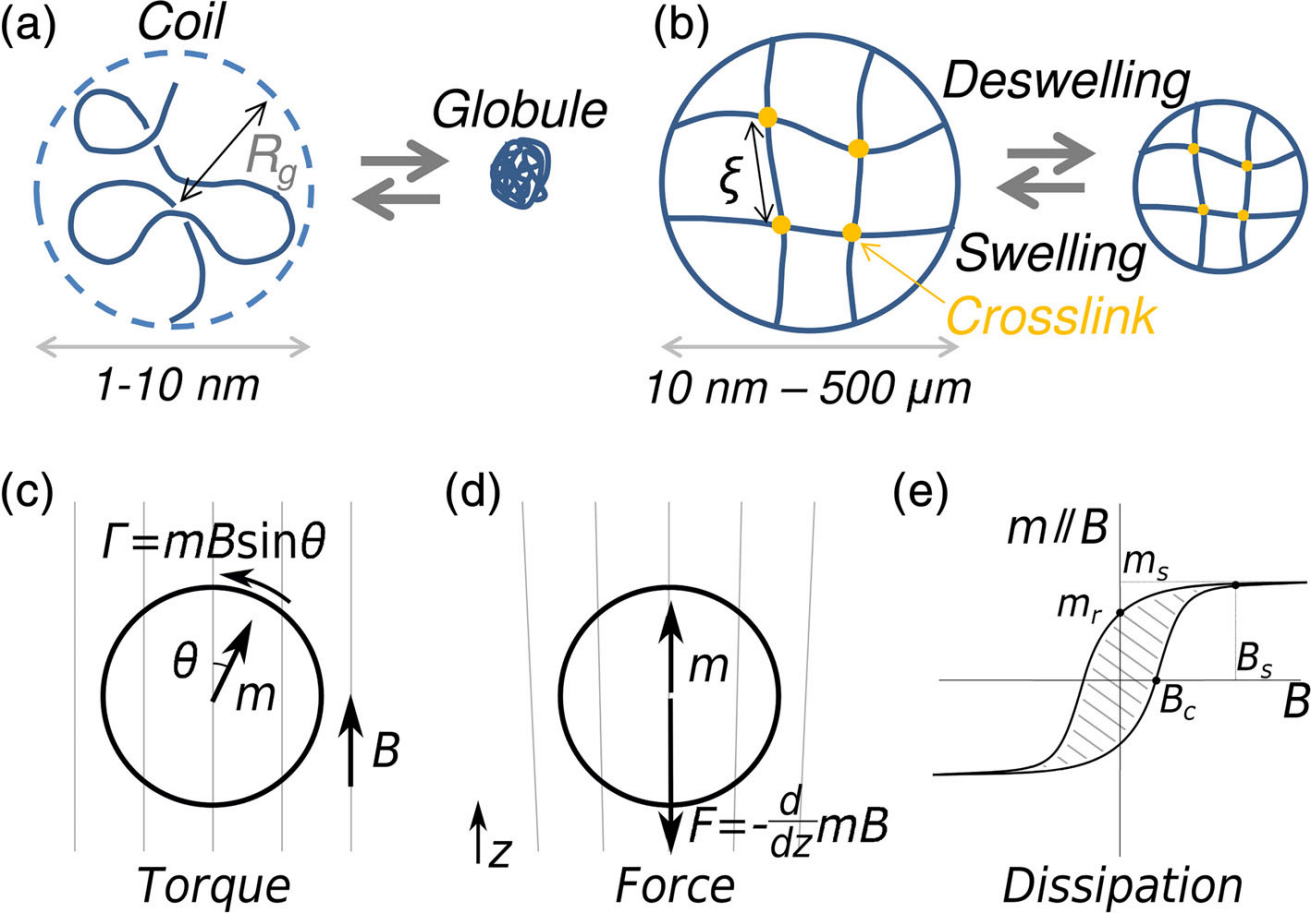
Analogy between (a) the coil‐globule transition of a single polymer chain and (b) the volume phase transition of a polymer gel particle. *R*_*g*_ and *ξ* denote the radius of gyration and mesh size, respectively. (c) Magnetic nanoparticle (MNP) in a uniform field experiences a torque and (d) in a nonuniform field, there is an additional force. (e) In a hysteresis loop, we plot the component of the magnetic moment in the MNP parallel to the field. In a zero field, the MNP may have a remanent component parallel to the field (*m*_*r*_). The field at which this component is zero is called the coercivity (*B*_*c*_). The saturation field is defined as the point where the hysteresis loop closes (*B*_*s*_). The area inside the loop is proportional to the energy dissipated in one field cycle

Linear polymers dissolved in a good solvent can be considered as a random coil.^9^, ^10^ The term “random coil” indicates that all monomers undergo Brownian motion with the constraint that they are connected to one another to form a long linear chain. However, for stimuli‐responsive polymers, this random coil configuration can abruptly transform into a globule (Figure 2(a)) in response to a small change in environmental conditions.^11^ In principle, this transition is a reversible first‐order phase transition,^11^ and many biological macromolecules show this transition in aqueous solutions. For example, protein chains can be folded into globules with spatial tertiary structures.^12^ Long DNA chains can also collapse into condensed nanoscale objects because of macromolecular crowding (i.e., depletion interaction^13^) or following the addition of multivalent cations (i.e., charge compensation^14^).

For synthetic macromolecules, poly(*N*‐isopropyl acrylamide) (PNIPAM) is the most studied polymer, and it shows a well‐characterized coil‐globule transition around a critical temperature.^15^, ^16^ This transition results from augmented hydrophobicity when the temperature exceeds the lower critical solution temperature (LCST). The LCST of PNIPAM is typically around 32°C, but it can be tuned to be above 37°C by varying the monomer composition (i.e., by incorporating more hydrophilic moieties). Apart from temperature sensitivity, pH‐responsivity can be induced in a PNIPAM chain through copolymerization with ionizable monomers; however, its effects on the LCST should be considered before it is induced. For example, the copolymerization of pH‐responsive monomers can be designed to generate charge–charge repulsion (thus resulting in the gel swelling), which is highly sensitive to the changes in ionic microenvironment around the physiological pH (~7.4) at body temperature.^17^, ^18^ Such synthesis strategies render PNIPAM more suitable for bioengineering applications, such as local hyperthermia therapeutics or pH‐responsive smart drug release. In contrast to the LCST‐type thermoresponsive polymers, there is another type of synthetic polymers that show a globule‐to‐coil transition when the temperature exceeds a critical point, which is termed upper critical solution temperature (UCST). However, the extent of use of UCST‐type thermoresponsive polymers in biomedical science has been considerably lower than that of LCST‐type polymers.^19^, ^20^

When individual hydrophilic polymer chains are cross‐linked together to form a swollen three‐dimensional (3D) network, the resulting material is called a hydrogel.^10^, ^21^, ^22^ The theoretical background of stimuli‐responsive hydrogel systems has been summarized in the review paper of Koetting et al.^23^ In principle, the physical concepts pertaining to polymer solutions are applicable to hydrogel systems since the major constituent of a hydrogel is an aqueous solvent (around 90% by volume for hydrogels commonly used for biomedical purposes) containing dissolved polymers.^24^

While hydrogels are liquid‐like solutions in which water‐dissolved polymer chains tend to maintain maximum spatial distribution to minimize the mixing free energy (i.e., maximizing the mixing entropy), they are also solid‐like since the cross‐linked polymer matrix is elastic. They retain their elastic free energy since they swell, but they do not fully dissolve in aqueous environments because of the cross‐linked polymer networks and maintain their 3D structures.^25^ In the case of tissue‐derived biopolymer hydrogels, such characteristics render physicochemical microenvironments similar to those of native extracellular matrices.^26^, ^27^, ^28^ In addition, the elasticity of swollen biopolymer hydrogels generally matches that of connective tissues,^29^, ^30^ which makes them biocompatible, and they can be adequately used in cell therapy and for tissue regeneration.

Generally, for a hydrogel in a swollen state, the balance between polymer–water mixing free energy and the elastic free energy of the cross‐linked polymer network determines the dimensions of the hydrogel at equilibrium. If hydrogel‐containing polymers are responsive to external stimuli, the hydrogel can exhibit a phase transition by swelling and deswelling (shrinking)^31^ since each water‐exposed polymer segment between cross‐links can undergo a coil‐globule transition.^32^, ^33^ Such a phase transition of a hydrogel is called volume phase transition, and it is a macroscopic analogue of the coil‐globule transition of a single polymer chain (Figure 2(a),(b); ^25^, ^34^). In fact, the swelling of a hydrogel can be regarded as the diffusion of locally concentrated polymers in solution to maximize the mixing entropy. Since the polymers cross‐link with each other, the polymer network can be simplified as an elastic continuum. Consequently, the hydrogel swelling can be phenomenologically considered as the collective (cooperative) diffusion of polymers.^25^, ^35^ Typically, the collective relaxation time is proportional to the square of the characteristic length of the gel, and therefore, smaller hydrogels swell faster.^36^ It should be noted that the different dimensional scales of the miniaturized gels may range from 50 nm to 500 μm for MGs, and from 10 nm to 1 μm for NGs, depending on the synthetic strategies and biological environments. This rapid responsivity is one of the main advantages of MGs and NGs. The stimuli‐sensitive swelling–deswelling volume phase transition of microscale hydrogels (i.e., MGs and NGs) has been widely used in biomaterial and pharmaceutical technology.^36^, ^37^, ^38^, ^39^, ^40^

### Diffusion and transport phenomena

Diffusion and convection are core mechanisms responsible for the use of hydrogels in biomedical applications, especially for controlled drug release.^41^ Drug delivery systems can have two possible distinguishable diffusion mechanisms, one corresponding to a static hydrogel network and the other to a dynamic hydrogel network. In a swollen sate, thermodynamics of solute transport is coupled with the elasticity and swelling behavior of the polymer chain network.^42^ In particular, for magnetic drug delivery, the convective transport plays a dominant role in the processes of magnetically actuated drug release kinetics.

#### Diffusion‐limited transport

We first consider diffusion‐controlled delivery^43^ for the case in which the hydrogel network remains static with time and the drug release rate is governed only by molecular diffusion.^44^ There are two types of diffusion‐controlled release systems: (a) the reservoir type and (b) the matrix type. In the former, a drug reservoir is embedded in or encapsulated by the hydrogel matrices or layers. Since the reservoir serves as a constant source, the drug concentration gradient is time‐independent, resulting in a steady‐state flux of drug molecules from the hydrogel to the exterior.^45^ In such a situation, Fick's first law suffices to quantify the drug release characteristics. In the matrix‐type release system, a finite amount of drugs is homogeneously distributed throughout the hydrogel matrix. Therefore, the flux of drug molecules to the exterior changes with time, implying that Fick's second law should be applied to capture the kinetics of the drug diffusion process.^44^

#### Dynamic hydrogel networks

The second case is a swelling‐controlled release system, which refers to a dynamically swelling hydrogel matrix in which drugs are uniformly dispersed.^44^ In this case, the hydrogel has a moving boundary, in contrast to the matrix‐type diffusion‐controlled release system, which has a stationary boundary. If the deswelling controls the drug release processes, the physical mechanism can be described by the convection‐diffusion equation.^46^ Convective transport, together with diffusion, is a major feature of the physical transport phenomenon in smart hydrogel systems.^47^ Compared with diffusion‐controlled release, externally forced convective transport facilitates significantly faster and active delivery of drug molecules.^48^

#### Convective transport in magnetic drug delivery

Convective transport phenomena are strongly implicated in studies of the magnetic drug delivery in vivo. First, the body‐injected magnetic nanodrug carriers are subjected to a blood or lymph flow through the peripheral microvascultures.^49^, ^50^, ^51^, ^52^, ^53^ In order to enable magnetic targeting, this convective force should be overcome by the locally focused static magnetic fields. Accordingly, the design of magnetic drug delivery systems has been performed under the consideration of the (a) size, position, geometry, and field strength of magnet; (b) the size, surface characteristics, and magnetic properties of the drug carriers; (c) the structure and dimensions of microvessels; and (d) the physiologically relevant blood flow velocity.

Second, instead of depositing drug carriers at a target site using a static magnetic force, wireless electromagnetic manipulation, and control of the motion of colloidal micropropellers by applying rotating magnetic fields can promote the transport of nanodrug carriers via augmented local fluid convection.^54^, ^55^ This newly emerging approach is an alternative to the invasive convection‐enhanced drug delivery platforms,^56^ but needs to be further investigated based on the in vivo animal models.

Finally, magnetic field‐induced actuation of drug‐loaded ferrogels has been highlighted as an efficient method for on‐demand and remotely controllable drug delivery devices.^57^ Magnetically responsive and reversible ferrogel volume change can be achieved by tailoring the spatial distribution of MNPs arrested in an elastic hydrogel, or by controlling the structural inhomogeneity of the gel matrix itself.^58^, ^59^, ^60^ Those designs may result in a bending, twisting, or shrinking of the ferrogel upon application of external magnetic fields. The convective mass flux of payload molecules, which is driven by the volumetric deformation of the ferrogels upon magnetic stimulation, was shown to enable controlled pharmacokinetics and dynamic release patterns.^61^

### Magnetic nanoparticles

Since magnetic fields permeate bulk tissues and organs, MNPs have a strong advantage over other types of inorganic biomaterials in biomedical applications.^62^ MNPs in the body can be remotely manipulated by external magnetic fields and guided to target tissue loci in vivo.^63^, ^64^, ^65^ When alternating magnetic fields are applied, thermal energy is generated in the MNPs owing to Néel and Brownian relaxations or hysteresis losses. Therefore, NPs can be used as a field receptor and energy transducer in biological environments. MNPs are also used as contrast agents in medical imaging, either because they produce local magnetic fields that change the contrast in magnetic resonance imaging (MRI) or because they can be detected in magnetic particle imaging (MPI).

Excellent reviews of the general properties of MNPs^66^, ^67^ and their applications in biomedical fields^68^, ^69^, ^70^, ^71^ exist in the literature. In the following, we summarize the physical properties of MNPs, with emphasis on the use of MNPs in MGs and NGs.

#### Macrodipole and magnetic torque/force

In a discussion of MNPs, it is instructive to treat them as a magnetic macrodipole; we ignore the detailed distribution of atomic spins in the NPs and consider an entire NP as a magnetic dipole with dipole moment m (ampere meter square), which is the sum of the projection of all atomic spins onto the mean direction. The dipole moment can be a function of the applied field B (tesla) and generally increases with the NP size. Such a macrodipole experiences a torque in a uniform field, and an additional force in a nonuniform magnetic field (Figure 2(c),(d)).

#### Hysteresis loop and magnetic heating

The atomic spins in the MNP might have an easy axis, namely, a preferred direction of magnetization, which depends on the atomic crystal lattice and the MNP shape. Such an easy axis may cause remanence; in other words, the magnetic moment of the macrodipole does not fall to zero when the field is removed. The easy axis may also cause hysteresis (i.e., the magnetic moment of the MNP depends on the history of the field applied). Figure 2(e) shows the projection of the magnetic moment in the direction of the applied field, as a function of the field intensity. When the field is zero, the component of the magnetic moment in the direction of the field does not drop to zero, but has a finite remanent value *m*_*r*_. When the field is swept, hysteresis leads to energy loss and heating of the MNP. The energy loss is proportional to the area indicated by diagonal lines in Figure 2(e).

#### Zero remanence, superparamagnetism, and magnetic domains

In most applications, it is desirable that MNPs have minimal remanence. In such a case, the MNPs do not attract each other when there is no external field, and it is convenient to use them in the form of a suspension. Since only MNPs with an easy axis show remanence, it is important to produce spherical MNPs. It is difficult to avoid a preferred direction of magnetization, which is intrinsic to atomic crystals. There are methods to suppress remanence, using superparamagnetism and magnetic domains (see Supplementary Information).

#### Néel and Brownian heating

When the field is swept, the atomic spins in the NP can follow either through the rotation of the entire MNP by a torque or because of the rotation of the spins in the MNPs.^72^ The rotation of the MNP is relatively slow (Brownian relaxation), while the rotation of the spins in the MNP can be very fast (Néel relaxation).

Although the start and end situations are identical for Brownian or Néel relaxation, the spin distributions in relation to the applied field, and therefore the energy loss, may be differ between these two situations. In the case of pure Brownian relaxation, the loss is *2mB* (torque integrated over angle). In the case of pure Néel relaxation, the maximum loss is *4mB*_*c*_ (maximum area under the hysteresis loop). Since Néel relaxation is considerably faster than Brownian relaxation, the Néel loss is the maximum loss. Applying fields higher than the saturation field (*B*_*s*_ in Figure 2(e)) does not increase the loss.

For nonremanent MNPs, the relation between the moment and the field around *B* = 0 can be linearized. In this approximation, Brownian losses, and also Néel energy losses (not discussed here), are proportional to *B* ^2^.^73^ The total power dissipated in the MNPs is proportional to the frequency of the field (*B* ^*2*^ *f*).

### Magnetic microgels and nanogels

#### Miniaturized magnetic gels

Magnetic hydrogels can be formed by confining water‐based suspensions of magnetic particles to the swollen 3D network of polymers.^74^, ^75^ The MNPs render the elastic hydrogel matrices magnetic field‐sensitive, apart from enhancing the polymer‐originated temperature‐ or pH‐responsivity of the gel network.^76^, ^77^ By combining the advantages of intelligent hydrogels and MNPs, we can devise a robust method to fabricate smart biomaterials that respond to minute changes in tissue microenvironments and that can be controlled externally through magnetic fields.^78^, ^79^ Such hybrid materials can be miniaturized on MGs and NGs for use as soft particulate biodevices.^80^ Through the application of surface functionalization techniques, these devices can be made to respond rapidly to multiple stimuli.^81^ These hybrid materials can be prepared with sizes comparable to those of cells (10–20 μm), organelles (1–5 μm), or biomacromolecular complexes (10–500 nm).^82^ A polymeric gel matrix has cytocompatible surface characteristics and tissue‐compatible mechanical properties. Therefore, they can in principle be adequately utilized as in vivo circulating nanohybrids that automatically respond to ambient physicochemical conditions and that can be spatiotemporally manipulated through external magnetic fields.

#### Design of magnetic microgels and nanogels

**FIGURE 3:**
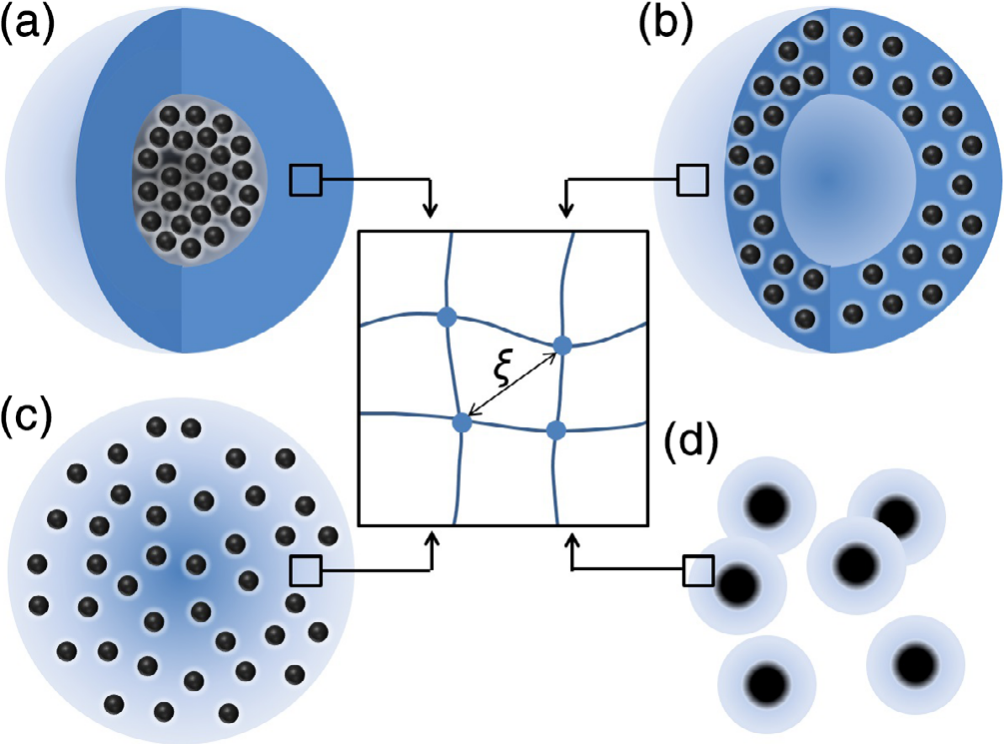
Hybridization modes of magnetic microgels and nanogels. (a) A microgel composed of a hydrogel shell with a magnetic core. (b) A microgel comprising a magnetic hydrogel shell that is empty at the center, or a microgel coated with an outer layer of magnetic nanoparticles (MNPs). (c) A magnetic microgel with homogeneously embedded MNPs. (d) A nanogel with a hydrogel‐coated MNP core (*ξ*: hydrogel mesh size)

Depending on the gel‐MNP hybridization type, MMGs and MNGs may fall into one of the following categories (Figure 3):

1. Hollow gel‐shells filled with magnetic fluids^83^: Compact core–shell MGs^84^, ^85^, ^86^ can be classified in this category since the MNP core may be regarded as a high‐density magnetic fluid surrounded by a gel shell.
2. MGs or NGs with an MNP‐embedded or coated outer layer^87^: This category includes multilayered MGs fabricated using layer‐by‐layer deposition techniques.^88^
3. MGs embedded with MNPs^89^, ^90^, ^91^, ^92^, ^93^, ^94^, ^95^, ^96^: In this category, MNPs are physically entrapped in the gel matrix or are chemically conjugated to the polymer network. Furthermore, the gel network can be used as a template for MNP growth or MNPs and gel particles can be cosynthesized in situ.
4. Single MNPs coated with a hydrogel layer^85^, ^97^, ^98^: This category may include a large number of current MNGs used for biomedical applications. This structure minimizes the hybrid particle size, and therefore, it is generally used for applications where the role of MNGs as MNPs is more critical than that as hydrogel particles.

During the designing of these hybrids,^99^ it should be noted that the physical characteristics of each of the components (hydrogel and MNPs) may influence those of the other, resulting in the components exhibiting properties different from their intrinsic properties. Confinement of magnetic fluids in a hydrogel matrix, especially when the MNPs are immobilized, hampers the ability of the MNPs to rotate, leading to a reduction in the MNPs' susceptibility at low fields. This reduces the torque and forces that can be applied, as well as their hysteresis loss. On the other way around, the stimuli‐responsive behavior of MGs and NGs can also be affected following their hybridization with MNPs. For example, the influence of hybridized MNPs on the polymer volume fraction in a gel particle and polymer–water interaction could reduce the ability of the gel to swell and deswell.

Another key aspect in the physical design of magnetic gel hybrids is the time scale of gel swelling and deswelling in response to the applied magnetic fields. In particular, for repeated volume phase transition of MNP‐laden thermoresponsive hydrogels,^100^, ^101^ it is important to optimize the kinetics of gel collapsing and reswelling, coupled to the magnetic heating rate and efficacy. In this case, the relaxation time required for the polymer chain reconfiguration in the absence of the alternating magnetic field could be a limiting factor for the pulsatile actuation performance of the hybrid gel devices.^102^

## PART II: BIOMEDICAL APPLICATIONS AND TRANSLATIONAL TECHNOLOGIES

In Part I, we presented the background theory for MGs, NGs, MNPs, and their hybrids. In the second part of this review, we summarize recent progress in the application of these hybrids in biomedical fields, including controlled drug release, cancer therapeutics, protein and gene delivery, and bioseparation and biocatalysis.

**FIGURE 4:**
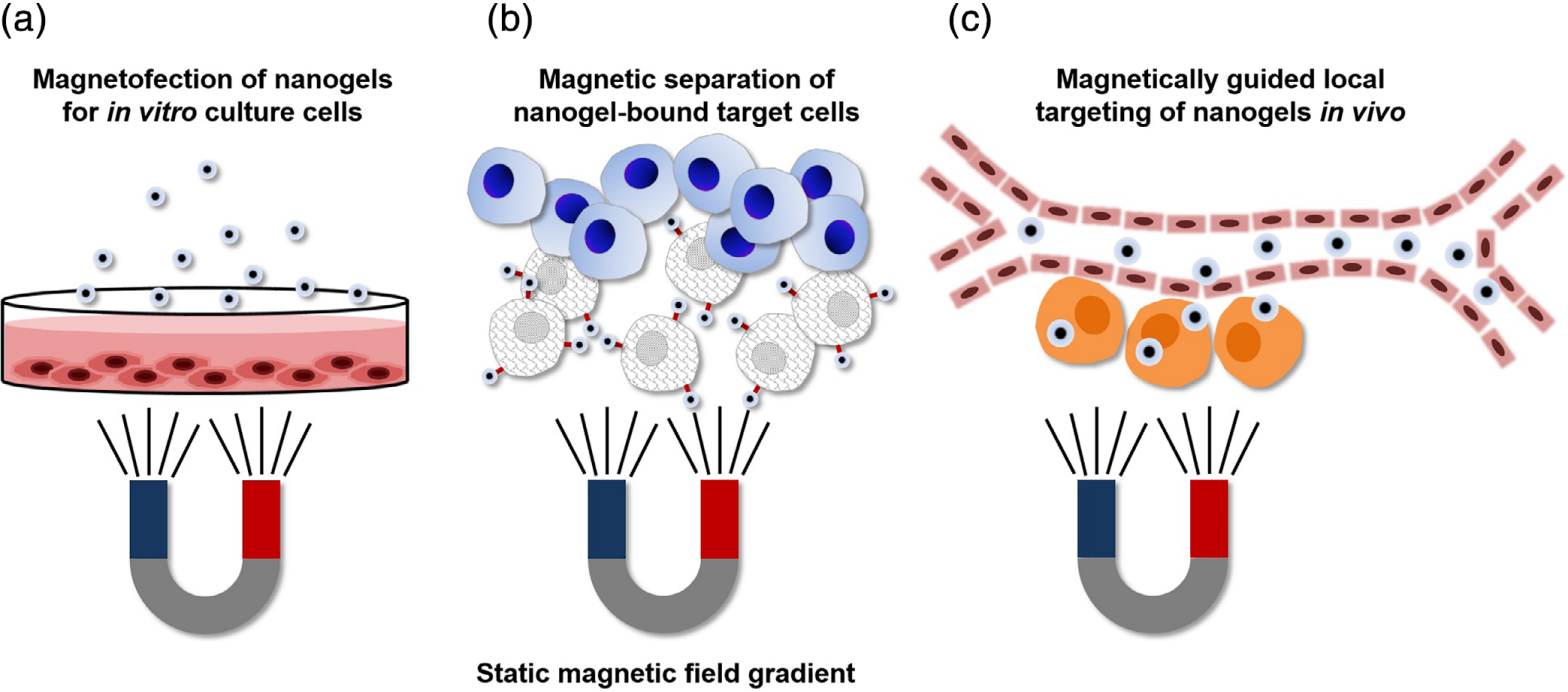
Biomedical application systems based on static inhomogeneous magnetic fields. (a) Magnetically driven nanogel infection (i.e., magnetofection) into in vitro culture cells. (b) Magnetic separation of nanogel‐bound target cells in aqueous media in vitro or in vivo. (c) Magnetic targeting of body‐injected nanogels to specific tissue loci

**FIGURE 5:**
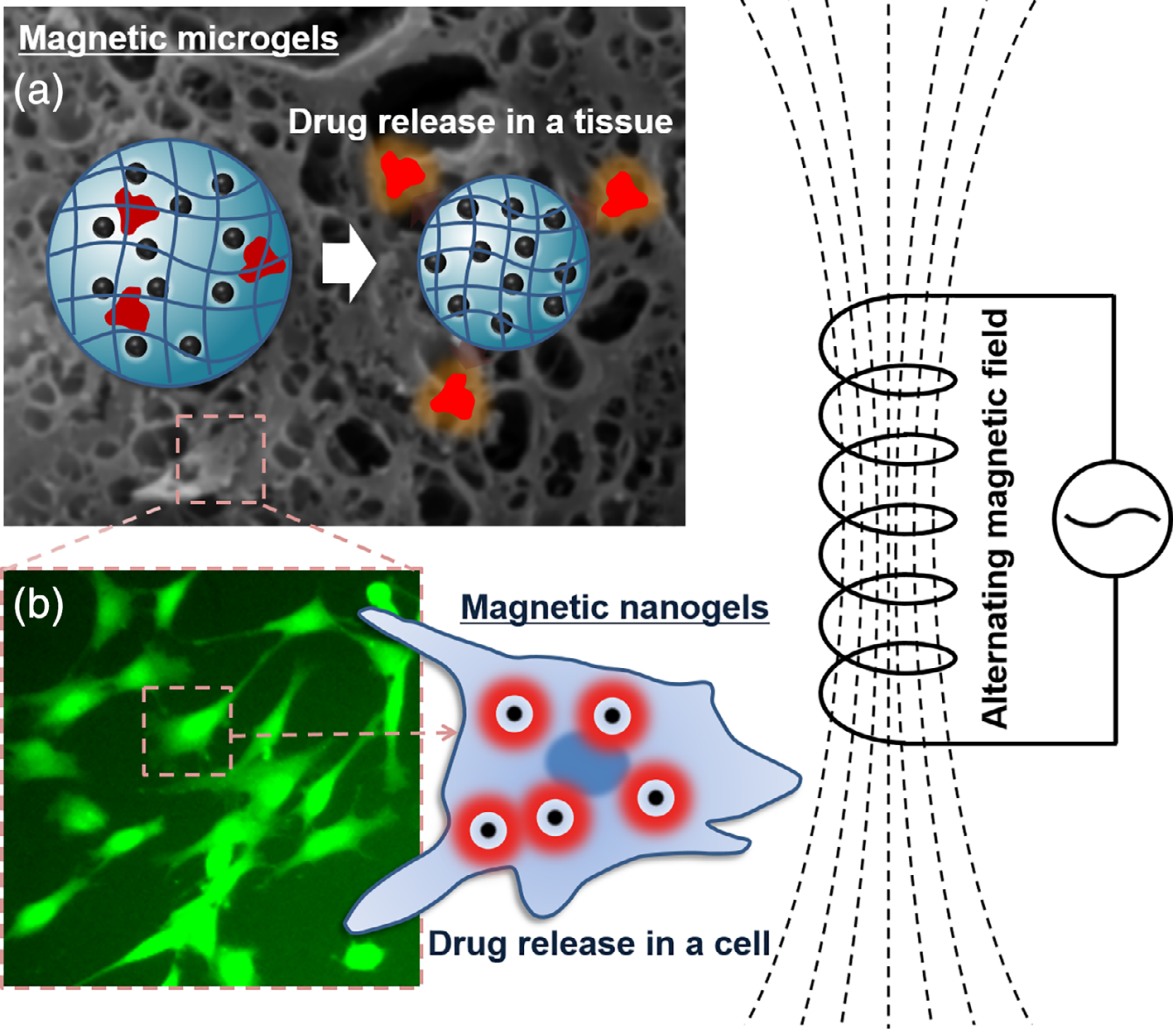
Biomedical application systems based on alternating magnetic fields. (a) Field‐responsive drug release from microgels at the tissue level. The background tissue image is from the authors' original cryoscanning electron microscopy data on an organ‐surface connective tissue structure (unpublished). (b) Field‐responsive drug release from nanogels at the cellular (or subcellular) level. The fluorescence microscopy image is from the authors' original data on the calcein‐stained culture of mesenchymal stem cells (unpublished)

There are various strategies to exploit the unique physical properties of MNP‐hybridized MGs/NGs in biomedical microdevices.^103^, ^104^ For diverse biophysicochemical interfaces,^105^ such hybrid biodevices are designed to have specific responsivity to target cell membranes or tissue microenvironments,^106^ mostly as injectable colloidal platforms.^107^ Functional combinations can be used to guide microdevices to a local region by generating gradients of static magnetic fields (Figure 4), and to increase the local temperature by applying alternating magnetic fields at a high frequency (Figure 5). These strategies are summarized in Tables 1, 2, 3 (and Tables S1–S3, Supplementary Information).

**TABLE 1:** Magnetic microgels and nanogels for drug delivery and hyperthermia

Size	Shape and structure	Polymeric materials	Magnetic materials	Magnetic properties	MNP hybridization method	Magnetic field controls	Applications	Reference
0.5–18 μm	Spheroid protein gel with embedded Fe_3_O_4_ NPs	Bovine hemoglobin	Coprecipitated Fe_3_O_4_ NP (20–30 nm in diameter)	Ferro‐ or ferri‐magnetic, *M*_s_ = 70–100 emu/g, coercivity (at 5 K) = 400–750 Oe	In situ coprecipitation of protein gels and MNPs upon addition of Fe^2+^	Static field application for separation in aqueous solutions	Temperature‐responsive drug release	114
~380 nm	Spherical multilayer core–shell of polymers and MNPs	Poly(*N*‐isopropylacrylamide)	Presynthesized iron oxide NP (~5 nm in diameter)	Superparamagnetic, *M*_s_ = 42–46 emu/g	Layer‐by‐layer assembly of core–shell structures	N/A	Thermoresponsive drug delivery	88
400 nm	Spherical Fe_3_O_4_ NP cluster coated with thermo‐ and pH‐sensitive polymer gel	Poly(*N*‐isopropylacrylamide) and chitosan	Presynthesized Fe_3_O_4_ NP (13 nm in diameter, *M*_s_ = 36 emu/g)	Superparamagnetic, *M*_s_ = 9 emu/g	Polymerization of gel layer on the MNP surface	N/A	Magnetically guided drug delivery	91
240–360 nm	Spherical polymer gel where MNPs are embedded	Copolymerized *N*‐vinylcaprolactam, acetoacetoxyethyl methacrylate, and vinylimidazole	In situ synthesized Fe_3_O_4_ NPs (~10 nm in diameter)	*M*_s_ < 30 emu/g	In situ synthesis of MNPs in the microgel templates	N/A	Magnetically controlled drug release	89
65–110 nm	Spherical polymer gel where MNPs are embedded	Poly(*N*‐isopropylacrylamide‐acrylic acid)	Presynthesized Fe_3_O_4_ NP (~15 nm in diameter)	N/A	Microgel synthesis in the presence of MNPs (ferrofluid)	Static field application for separation in aqueous solutions	Magnetically guided drug delivery	90
~10 μm	Magnetic core and polymeric gel shell	Hydroxyethyl starch‐hydroxyethyl methacrylate and hydroxyethyl starch‐polyethylene glycol methacrylate	Presynthesized Fe_3_O_4_ NP (77 nm in diameter)	Superparamagnetic	Microgel synthesis in the presence of MNPs (ferrofluid)	Magnetorelaxometry	Long‐term drug release by degradation	116
~294 nm	Spherical polymer gel where Fe_3_O_4_ NPs are embedded	Poly(N‐isopropyl acrylamide‐methacrylic acid‐hydroxy ethyl methacrylate)	Presynthesized iron oxide NP	*M*_s_ = 18 emu/g	Physical adsorption of MNPs in the nanogel matrix	N/A	Magnetic hyperthermia and pH‐ and thermo‐sensitive drug release	130
90–98 nm	Spherical MNP coated with thermo‐ and pH‐sensitive polymer gel	Polyallylamine and polyacrylic acid	Presynthesized Zn/co/Mn/Fe_2_O_4_ NP	Superparamagnetic, M_s_ = 13–46 emu/g	Nanogel shell synthesis in the presence of MNPs (ferrofluid)	High‐frequency alternating magnetic fields for triggering drug release	Alternating magnetic field‐induced drug release	131
90–260 nm	Spherical polymer gel where MNPs are embedded	Poly(acrylamide‐acrylic acid)	Magnetite (Fe_3_O_4_) (8–12 nm in diameter)	N/A	Microgel synthesis in the presence of MNPs (ferrofluid)	N/A	Drug delivery and magnetic hyperthermia	177

Abbreviations: MNP, magnetic nanoparticle; NP, nanoparticle.

**TABLE 2:** Magnetic microgels and nanogels for cancer therapies

Size	Shape and structure	Polymeric materials	Magnetic materials	Magnetic properties	MNP hybridization method	Magnetic field controls	Applications	Reference
<200 nm	Spherical Ni‐Ag NP coated with a pH‐responsive copolymer gel shell	Poly(ethylene glycol‐*co*‐methacrylic acid)	Magnetic Ni NPs on which fluorescent metallic Ag layer is grown (13–23 nm in diameter)	Ferromagnetic, *M*_s_ ~ 3 emu/g	Microgel synthesis in the presence of MNPs (ferrofluid)	pH‐dependent magnetic manipulation with static fields	pH‐responsive anticancer drug delivery and fluorescent pH‐sensing and imaging	121
110 nm	Ferrofluid‐encapsulating polymer shell	Poly(acrylic acid‐*co*‐distearin acrylate), poly(γ‐glutamic acid‐*co*‐γ‐glutamyl oxysuccinimide)‐*g*‐poly(ethylene glycol)‐folate, and chitosan	Presynthesized iron oxide NP	Superparamagnetic	Microgel shell synthesis in the presence of MNPs (ferrofluid)	MRI	Anti‐cancer drug delivery and MRI	127
100–143 nm	Ferrofluid‐encapsulating polymer shell	Thiolated alginate	Presynthesized iron oxide NP (16–26 nm in diameter)	Superparamagnetic, *M*_s_ ~ 41 emu/g	Nanogel shell synthesis in the presence of MNPs (ferrofluid)	N/A	Magnetically targeted anticancer drug delivery	123
~70 nm	Spherical au/Fe_3_O_4_ NP coated with thermo‐ and pH‐sensitive polymer gel	Poly(ethylene glycol)‐*b*‐poly((*N*,*N*‐dimethylamino)ethyl methacrylate‐*co*‐2‐hydroxyethyl methacrylate)‐maleic acid	Presynthesized Au/Fe_3_O_4_ NP	Superparamagnetic, *M*_s_ = 24 emu/g	Nanogel shell synthesis in the presence of MNPs (ferrofluid)	N/A	Magnetically targeted anticancer drug delivery	124
300 nm	Spherical Fe_3_O_4_ NP coated with thermo‐ and pH‐sensitive polymer gel	Folate‐ or *L*‐5‐methyltetrahydrofolate‐modified poly(ethyleneimine) and poly(*N*‐isopropyl acrylamide	Presynthesized iron oxide NP (10 nm in diameter)	N/A	Nanogel shell synthesis in the presence of MNPs (ferrofluid)	N/A	Magnetically targeted anticancer drug delivery	129
~170 nm	Magnetic core and polymeric gel shell	Poly(*N*‐isopropylacrylamide‐*co*‐acrylamide)	Presynthesized fluorescent iron oxide NP	Superparamagnetic, *M*_s_ ~ 5 emu/g	Nanogel shell synthesis in the presence of MNPs	Static field application for guidance in aqueous solutions	Magnetically targeted and thermo‐sensitive anticancer drug delivery with bio‐imaging	133
<200 nm	Spherical Ni‐Ag MNP coated with a pH‐sensitive copolymer gel shell	Poly(ethylene glycol‐*co*‐methacrylic acid)	Magnetic Ni NP on which fluorescent metallic Ag layer is grown (13–23 nm in diameter)	Ferromagnetic, *M*_s_ ~ 3 emu/g	Nanogel shell synthesis in the presence of MNPs	Static field application for pH‐dependent magnetic manipulation	pH‐responsive anticancer drug delivery with cell imaging	121
~90 nm	Spherical MNP coated with a thermo‐ and pH‐responsive copolymer gel shell	PEG‐*b*‐P(DMAEMA‐*co*‐HEMA)‐*g*‐PNIPAAm	Fe_3_O_4_/Ag NP (~50 nm in diameter)	Superparamagnetic, *M*_s_ = 24 emu/g	In situ polymerization of nanogel layer on the MNP surface	N/A	Thermo‐ and pH‐responsive anticancer drug release	124
> 98 nm	Spherical polymer gel where Fe_3_O_4_ NPs are embedded	P(NIPAM‐*co*‐DMAEMA‐*co*‐AFA)	Presynthesized Fe_3_O_4_ NP	*M*_s_ = 60 emu/g	Physical entrapment of MNPs by nanogel deswelling	N/A	Thermo‐ and pH‐responsive anticancer drug release	125
200–250 nm	Spherical polymer gel where MNPs are embedded	Chitosan‐*g*‐poly(*N*‐vinylcaprolactam)	Presynthesized Fe_3_O_4_ NP	*M*_s_ ~ 37 emu/g	Nanogel synthesis in the presence of MNPs (ferrofluid)	High‐frequency alternating magnetic fields for hyperthermia	pH‐responsive anticancer drug release	134
~120 nm	Cube‐like peptide gel where MNPs are entrapped	Fmoc‐Tyr(H_2_PO_3_)‐OH	Presynthesized zinc‐doped Fe_3_O_4_ NP	*M*_s_ = 84.3 emu/g	In situ polymerization of nanogel in the presence of MNPs	Alternating magnetic fields for hyperthermia	Thermoresponsive release of reactive oxygen species	135

Abbreviations: MNP, magnetic nanoparticle; NP, nanoparticle.

**TABLE 3:** Magnetic microgels and nanogels for protein/gene delivery/therapeutics and tissue engineering

Size	Shape and structure	Polymeric materials	Magnetic materials	Magnetic properties	MNP hybridization method	Magnetic field controls	Applications	Reference
5–20 μm	Spherical polymer gel where MNPs are embedded	Gelatin and poly(*N*‐isopropylacrylamide‐*co*‐acrylamide)	Presynthesized iron oxide NP	Superparamagnetic	Microgel synthesis in the presence of MNPs (ferrofluid)	High‐frequency alternating magnetic fields for triggering drug release	Magnetically triggered protein delivery for tissue engineering	150
190–250 nm	Magnetic core (clustered MNPs) and polymeric gel shell	Polyacrylamide	Presynthesized Fe_3_O_4_ NP	N/A	Nanogel synthesis in the presence of MNPs (ferrofluid)	Static field application for separation in aqueous solutions	Magnetically guided protein delivery and cellular metabolic manipulation	148
100–180 nm	Spherical polymer gel where MNPs are embedded	Cholesterol‐bearing pulluran	Presynthesized Fe_3_O_4_ NP (12 nm in diameter)	N/A	Physical adsorption of MNPs in the nanogel matrix	Static field application for magnetic guidance	Magnetically guided intracellular protein delivery	149
60–160 nm	Spherical polymer gel where MNPs are embedded	Adenine functionalized chitosan and thymine functionalized heparin	Presynthesized Fe_3_O_4_ NP	Superparamagnetic, *M*_s_ ~ 39 emu/g	Nanogel synthesis in the presence of MNPs (ferrofluid)	Static field application for magnetic guidance	Magnetically guided intracellular protein delivery for cartilage and bone regeneration	151
200–450 μm	Cubic polymer gel where MNPs are embedded	Gelatin methacrylate	Presynthesized iron (II, III) oxide nanopowder (<50 nm)	N/A	Microgel synthesis in a ferrofluid	Static field application for 3D cell scaffold assembly	Magnetic assembly of multilayer 3D tissue constructs	173
100–750 nm	Spherical polymer gel where MNPs are embedded	Polymerized ethyl acrylate, methacrylic acid, and di‐allyl phthalate	In situ synthesized iron oxide NPs (5 nm in diameter)	N/A	In situ synthesis of MNPs in the microgel templates	MRI	Stem cell labeling and tracking with MRI after transplantation	176
40–200 nm	Spherical polymer gel where MNPs are entrapped	2‐Vinylpiridin and divinylbenzene	Presynthesized iron oxide nanocrystals (7 nm in diameter)	N/A	Physical entrapment of MNPs by nanogel deswelling	N/A	Gene delivery and magnetic hyperthermia	152
230–250 nm	Spherical amphiphilic gel complexed with exosomes	Cholesterol‐bearing pullulan	Presynthesized Fe_3_O_4_ NP	N/A	Nanogel synthesis in a ferrofluid	Static field application for magnetic guidance	Magnetically assisted intracellular RNA delivery for neural cell differentiation	154

Abbreviations: MNP, magnetic nanoparticle; NP, nanoparticle.

Let us consider possible scenarios for such platforms. After injection into the body, drug‐loaded MMGs and MNGs can be guided by magnetic forces to specific locations in vivo.^53^ Subsequently, depending on the gel responsivity, the MG/NG can swell or shrink when the local temperature or pH changes, and release the drugs in a pulsatile manner. In the case of thermoresponsive gels, hybridized MNPs can be used as components that generate heat in response to external alternating magnetic fields. Alternatively, the gel polymers can be biodegraded by enzymes in vivo, resulting in the gradual release of drug molecules. If the gel surfaces are functionalized with specific ligands, the gel particles may selectively interact with certain types of cells. Apart from heating, the MNP component in gel particles can be located through MRI or MPI. Since the MNPs may trigger various toxicological pathways in vivo,^108^ predominantly via intracellular oxidative stress, careful optimizations in MNP surface biocompatibility and core biodegradability are required for the colloidal platform design.^109^ In addition, topical delivery of NG‐based therapeutics can generate reactive oxygen species or induce DNA damage, when the NGs are captured by keratinocytes in the human skin.^110^ These adverse effects of NGs and MNPs need to be further analyzed particularly focused on the long‐term toxicological outcomes.^110^

Drug delivery is currently the main application area for MMGs and MNGs. However, they can be applied in many other important frontier fields in precision medicine, such as cancer chemotherapy, protein and gene therapeutics, tissue engineering, and regenerative medicine, apart from bioseparation and biocatalysis. Notably, all these applications are mutually related in terms of engineering principles and practical uses. In tissue engineering, for instance, one may regenerate completely different complex tissue architectures depending on the extent of spatiotemporal control of the morphogen concentration within in vitro cellular architectures or in vivo native tissues provided by the drug delivery technique employed.^111^ The subject of drug delivery has a close relationship with gene delivery,^112^ strengthening multifaceted and cross‐related aspects of clinical applications of hydrogel‐based devices.

### Drug delivery for general purposes

There have been several physicochemical investigations on MMGs and MNGs with potential for a wide range of drug delivery and controlled release applications. These studies focused on synthesis, structure characterization, and stimuli‐controlled responsivity with or without the consideration of model drug release effectiveness.^113^, ^114^ The studies include investigations of temperature‐responsive volume phase transition^84^, ^88^ and subsequent magnetic heating,^91^ pH‐sensitivity with or without temperature‐induced transition^89^ and with static‐field guidance,^90^, ^92^, ^115^ and magnetization response to an applied static field.^93^, ^95^

For example, it has been reported that magnetite (Fe_3_O_4_) NP‐containing mesoporous silica particles coated with a gel of NIPAM and *N*‐hydroxymethyl acrylamide copolymer act as thermosensitive microspheres.^85^ By using Zn(II) phthalocyanine tetrasulfonic acid, a drug used in photodynamic therapy, the same study showed that the magnetic microspheres could be used for controlled drug release. An MG‐related study revealed that poly(vinyl alcohol)‐PNIPAM spheres containing iron oxide NPs could be guided by static magnetic fields and could release a diagnostic dye (congo red) when triggered by the field in a controlled manner.^96^ Additionally, Wöhl‐Bruhn et al.^116^ studied the magnetorelaxometry of a hydroxyethylstarch MG containing MNPs and its long‐term degradation release of fluorescein isothiocyanate‐labeled dextran.

An interesting numerical simulation study on the drug release mechanism was performed by Masoud and Alexeev.^83^ Using a coarse‐grained method, they computationally simulated the drug release characteristics of responsive MG capsules containing rigid microrods during swelling and deswelling. For the swelling process, the drug macromolecules were released through steady‐state diffusion, whereas the deswelling of the MG resulted in a burst of drug discharge because of the hydrodynamic transport of the solutes. The pore size change of the cross‐linked polymer matrix was correlated with the swelling and deswelling.

Another intriguing approach for the MMG/MNG‐based drug delivery is to embed PNIPAM NGs with Fe_3_O_4_ NPs in a networked ethyl cellulose membrane that is in contact with a drug reservoir.^117^, ^118^ Here, the embedded MNPs act as switch valves that are responsive to the external alternating magnetic field. In other words, if the alternating field induces heat generation in the superparamagnetic NPs, the PNIPAM NGs deswell and induce a flux of drug molecules from the reservoir. Rat subcutaneous transplantation experiments involving such nanocomposite membrane platforms have shown the possibility of long‐term on‐demand release of drugs for pain treatment, chemotherapy, and insulin delivery.^117^

Static fields can also be utilized for controlled delivery with transplantable MNP‐conjugated hydrogels. Zhao et al.^58^ developed a ferrogel platform in which iron oxide NPs were conjugated with a network of alginate hydrogels. Under application of a field, the ferrogel shrunk and was deformed, and it actively released model drugs and cells. In cell culture and mouse experiments, on‐demand delivery of mitoxantrone, plasmid DNA, chemokine molecules, and mesenchymal stem cells has been successfully achieved. This implies that such a ferrogel platform can not only be widely used for drug or cell delivery but also serve as an active scaffold for cells.

### Anticancer treatments

There have been attempts to use pH‐sensitive or near‐infrared‐light‐responsive MNGs as controllable carriers of anticancer drugs (such as doxorubicin, methotrexate, and fluorouracil), and they have shown in vitro effectiveness for HUVEC and HT29 cell lines^119^ and HepG2 cells,^120^ mouse melanoma B16F10 cells,^121^ HeLa cells,^122^, ^123^ and human breast adenocarcinoma cell line MCF7.^124^ Some pH‐sensitive PNIPAM copolymer‐based MMG composites have also been considered for potential application in tumor therapeutics.^85^, ^125^

In particular, Sunderland et al.^126^ reported that MNGs based on a maghemite iron oxide core coated with a network of poly(ethylene glycol) [PEG]–polycarboxylate copolymers can be useful for the magnetic separation of melanoma cells. They also showed that these MNGs could circulate in blood vessels with an extended time range in vivo. In addition, hollow NGs comprising acrylic acid and 2‐methacryloylethyl acrylate backbones grafted with PEG and PNIPAM have been engineered to contain superparamagnetic iron oxide NPs and anticancer agent doxorubicin.^127^ In this platform, the temperature‐ and pH‐responsive release of doxorubicin increased the anti‐HeLa cell toxicity. Guided by static magnetic fields to a local site, these NGs could be used as mediators for magnetically induced hyperthermia and as contrast agents for MRI.

High‐frequency magnetic field‐responsive PNIPAM‐based MGs have also shown controlled release capability for anticancer curcumin through induction heating of Fe_3_O_4_ NPs entrapped in the PNIPAM network.^128^ Furthermore, PNIPAM‐based MMGs that bind to the folate receptor of cancer cell membranes have been synthesized.^129^ These MGs have shown temperature and pH‐responsive volume changes, augmented loading and release efficiency at a high temperature (45°C) or a low pH (4.5), and high cellular uptake. Similar NG systems have been prepared by adopting an Au/ Fe_3_O_4_ core coated with PEG copolymer and PNIPAM gel layers for the delivery of methotrexate,^124^ by using Fe_3_O_4_ NPs with a layer of PNIPAM copolymer gel for cisplatin release,^130^ or by employing other magnetic materials and polymers.^131^ A naturally derived NG drug system has also been investigated.^132^

For the achievement of multifunctionality, a more complex hybrid NG platform was developed,^133^ and it comprised (1) a core cluster of Fe_3_O_4_ NPs with a porous carbon shell in which fluorescent carbon dots were embedded and (2) an outer PNIPAM NG layer in which drug (curcumin) molecules were loaded. This system exhibited responsivity caused by the heating of the superparamagnetic NPs by alternating magnetic fields and the heating of the carbon shell by near‐infrared light. Subsequent deswelling of the PNIPAM gel layer enabled drug release and photoluminescence tuning, leading to selective imaging and cytotoxicity of B16F10 cells in vitro. From a different perspective, another type of dual responsive MNGs was developed.^134^ Iron oxide NPs were embedded in a thermoresponsive chitosan‐*g*‐poly(*N*‐vinylcaprolactam) NG that was designed to release preloaded doxorubicin in response to high‐frequency magnetic fields. This NG platform showed synergic cytotoxicity for breast cancer cells for both hyperthermia and chemotherapy. A recent interesting study developed a neutrophil‐inspired magnetic peptide NG loaded with Fe_3_O_4_ NPs and chloroperoxidase.^135^ It demonstrated both in vitro and in vivo that the high‐frequency‐magnetic‐field‐stimulated MNPs could upregulate the H_2_O_2_ levels in cancer cells and that subsequently, chloroperoxidase converted H_2_O_2_ into singlet oxygen. This NG facilitated synergic anticancer therapy involving a combination of magnetic hyperthermia and enzymatic treatment.

### Protein therapeutics and gene therapy

The controlled delivery of proteins^136^, ^137^, ^138^, ^139^ and nucleic acids^140^, ^141^, ^142^, ^143^ through smart MG/NG platforms has recently attracted interest. DNA or RNA‐bearing NG particles have emerged as nonviral gene delivery vectors,^144^, ^145^, ^146^ as an alternative to viral gene delivery approaches that have limitations related to immunogenicity and loading capacity.^147^

For protein delivery, Lin et al.^148^ reported a magnetic enzyme NG platform based on an Fe_3_O_4_ core coated with a polyacrylamide gel network in which enzymes (lipase, peroxidase, trypsin, and cytochrome) were encapsulated. They showed the possibility of using the complex for static‐field controlled delivery of proteins and subsequent cellular metabolic manipulation. Another promising attempt involved a physically cross‐linked polysaccharide NG, “chaperone,” that was hybridized with oleic acid‐coated Fe_3_O_4_ NPs through hydrophobic interaction.^149^ When complexed with cargo model proteins (albumin and insulin), in vitro magnetofection tests showed effective protein delivery to target HeLa cell cytosols, indicating the potential application of the polysaccharide NG in protein therapeutics for cancer therapy.

Furthermore, a hybrid MG device that combines biodegradability and magnetic field‐responsivity has been developed.^150^ This MG comprised a noncytotoxically and covalently cross‐linked gelatin network in which thermoresponsive p(NIPAM‐*co*‐acrylamide) chains were trapped and ferrofluids were confined. Upon applying an alternating magnetic field, the hybridized gelatin matrix collapsed because of internal magnetothermal effects and the preloaded model drug (albumin) molecules were released through convective transport. On the basis of the biocompatibility and enzymatic degradability of this MG platform, potential applications in tissue engineering can be expected.

A nanoscale platform for in‐depth tissue engineering applications was developed by Fan et al.^151^ by utilizing nucleobase‐functionalized chitosan/heparin NG as a ferrofluid‐encapsulating scaffold and a protein delivery vector. The chemically modified biopolymers were assembled through base‐pairing hydrogen bonding to internalize Fe_3_O_4_ NPs, and the heparin component facilitated the adsorption of bone morphogenetic protein 2 (BMP‐2) with high efficiency. Guided by a static magnetic field in vitro, the biopolymeric MNGs promoted the human osteoblast MG‐63 viability by releasing the loaded cell growth factor BMP‐2. The protein release was considered to be triggered by enzymatically induced swelling and degradation. This is one more example of a biocompatible NG system that can be applied to cartilage and bone tissue regeneration.

The efficiency of intracellular delivery of nucleic acids can be significantly enhanced by using magnetic force. Among several in vitro studies, the experiment of Deka et al.^152^ is an interesting example. They prepared a pH‐responsive NG of 2‐vinylpyridine and divinylbenzene loaded with iron oxide NPs and short oligonucleotides. The NG could release DNA fragments under a pH change. In another intriguing result, Zhang et al.^86^ reported zwitterionic NGs of polycarboxybetaine methacrylate containing a core of Fe_3_O_4_ NPs that could be degraded for renal clearance. The model drug dextran was loaded in these MNGs. In cell culture experiments, the ligand‐conjugated MNGs showed significant cellular uptake by human umbilical vein endothelial cells, while macrophage uptake was low. Their possible use for the delivery of DNA and small interfering RNA (siRNA) was discussed. Alternatively, a hybrid MNG based on 2‐vynylpyridine‐*co*‐divinylbenzene copolymer could possibly be used for pH‐sensitive delivery of siRNA molecules.^153^

A recent study of Mizuta et al.^154^ demonstrated that MNG‐exosome complexes could improve the delivery efficiency of mRNA and microRNA (miRNA) under magnetic field guidance for controlling target cellular functions in vitro. Delivery vector hybridization was performed through hydrophobic fusion between a Fe_3_O_4_ NP‐containing amphiphilic NG and a pheochromocytoma PC12 cell‐derived exosome loaded with mRNA and miRNA. This nanohybrid RNA vehicle could be efficiently delivered into adipose‐derived mesenchymal stem cells under a static magnetic field to induce cellular differentiation in neuron‐like cells.

### Bioseparation and biocatalysis

Hydrogel composites have been extensively investigated for application to bioseparation^155^, ^156^ and biocatalysis^157^; these two terms refer to the selective separation of organic compounds from biologically relevant mixtures and the catalytic biochemical transformations usually related to enzymatic reactions. Owing to their large surface‐to‐volume ratio and high accessibility to surrounding aqueous milieu, miniaturized and chemically conjugated hydrogel beads can show strong functionality and responsivity when coupled with MNPs.

For enzyme immobilization and magnetic separation through static fields, Hong et al.^158^ reported Fe_2_O_4_ NPs coated with a polyacrylamide gel layer whose surface was functionalized with amine groups. By using this platform, they covalently immobilized a digestive proteolytic enzyme (α‐chymotrypsin). This showed the potential of the platform for use in the magnetic separation of enzymes. Another type of magnetic adsorbent MG for high‐efficiency protein separation under high‐gradient static fields was developed by Turcu et al.^159^ The MG was based on a hydrophobic core of clustered Fe_3_O_4_ NPs coated with a hydrogel shell of polyacrylic acid, poly‐*N*‐isopropopylacrylamide, and poly‐3‐acrylamidopropyl trimethylammonium chloride. The work of Ghaemy and Naseri^160^ showed that temperature‐ and pH‐responsive chitosan NGs whose surfaces were conjugated with Fe_3_O_4_ NPs could release a nonsteroidal anti‐inflammatory agent (sodium diclofenac) in a controlled manner. These hybrid NGs showed potential for use in magnetically assisted separation of proteins such as albumin.

In a different concept, Lin et al.^161^ reported a poly(ether sulfone) hydrogel membrane embedded with PNIPAM NGs and Fe_3_O_4_/Fe_2_O_3_ NPs. Under alternating magnetic fields, this hybrid hydrogel membrane was shown to function as a switchable biomacromolecular filter. In this system, the deswelling PNIPAM NGs acted as magnetoresponsive valves for dextran separation, which was triggered by induction heating of the coembedded magnetic NPs.

Recently, MMG‐based isolation of breast cancer cells in the whole blood was demonstrated by Seyfoori et al.^162^ An immune cell‐mimetic MG was prepared from the matrix of a PNIPAM copolymer in which Fe_3_O_4_ NPs were synthesized in situ. The gel surface was functionalized by cancer cell‐specific antibodies to capture the cells in a blood specimen and separate them under a local magnetic field. In another study, a clay MG containing iron oxide NPs and bacterial plasmids was developed for repeatable cell‐free protein synthesis and magnetic separation.^163^

The integration of stimuli‐responsive MGs with a magnetic biomanipulation system is another recent advancement. A gold‐nanorod‐embedded double network of alginate and PNIPAM was used to fabricate a near‐infrared‐responsive MG, which was incorporated as a mechanically functional component in a magnetic microgripper.^164^ This soft robot gripper first memorized the shape of a target microtissue and acted as a remotely controllable gripper through reversible thermoresponsive swelling in a physiological condition.

For biocatalysis, Lin et al.^148^ synthesized an MNG system by conjugating an Fe_3_O_4_ NP surface with oppositely charged enzymes. This MNP‐enzyme complex was subsequently coated with a cross‐linked polyacrylamide gel layer. Enzymes of various biological origins (i.e., *Candida rugose* lipase, horseradish peroxidase, trypsin, and cytochrome C) were tested for intragel encapsulation, and a wide range of magnetic field‐assisted nanobiocatalytic applications was expected, in addition to applications to protein delivery and cellular metabolic manipulation.

Wang et al.^165^ developed a novel hybrid NG probe in which superparamagnetic Fe_3_O_4_ NPs and dual enzymes (catalase and superoxide dismutase) were incorporated in a glycol chitosan gel matrix. This NG acted as a dual‐modality probe for (1) MRI by means of the superparamagnetic NPs and for (2) ultrasound imaging owing to O_2_ bubble generation by an enzymatic reaction with reactive oxygen species. These features facilitated the biocatalytic sensing of pathological microenvironments in vivo. Along the same lines, Wu et al.^166^ reported a magnetic core–shell MG platform in which Fe_3_O_4_ NPs were coated through free‐radical polymerization triggered by the cascade reaction of glucose oxidase and horseradish peroxidase. The encapsulated bienzyme components were covalently immobilized in the gel network, minimizing the diffusive dissipation of intermediate hydrogen peroxide between both the enzymes, which maximized the reaction efficiency. This system was designed to be suitable for colorimetric glucose sensing.

In addition to the above studies, there have been other investigations on these topics, and their results may have the potential for use for health and hygiene purposes.^167^, ^168^

### Regenerative medicine

Tissue regeneration is one of the most promising frontiers of MMG research. Over the past two decades, a variety of functional MGs and NGs have been developed for use in regenerative medicine.^169^, ^170^ For instance, mesenchymal‐stem‐cell‐encapsulating MGs could not only provide well‐defined biophysical and biochemical cues to predict the embedded cell's fate but also act as cell vehicles for targeted cell therapy.^26^, ^30^ In addition, the use of polymeric NGs in tissue engineering has mainly been focused on targeted in vivo imaging and cell migration tracing. Several studies have demonstrated that their performance in tissue engineering can be considerably improved when magnetic controllability is provided to those NG platforms. One of the advantages of providing magnetic controllability is that magnetic forces can be employed to assemble MG building blocks into complex 3D tissue constructs, which better mimic native tissues and organs in the body. Locally focused static fields can be used to precisely manipulate fibroblast‐seeded multicomponent MMG units in a programmable manner,^171^, ^172^ resulting in the realization of structured and heterogeneous tissue constructs. Another magnetic bottom‐up assembly method for cell‐embedded MGs has been developed by adopting layer‐by‐layer deposition around a magnet tip.^173^, ^174^ The loaded fibroblasts in 3D gel assemblies show guided growth and proliferation, depending on the gel mechanical properties and the coloaded biochemical.

In addition to field‐guided artificial tissue construction, self‐assembly of mesenchymal‐stem‐cell‐laden alginate MGs has been achieved through surface modification with binding pair molecules, such as biotin and streptavidin.^175^ Selective magnetic separation has been performed by introducing Fe_2_O_3_‐NP‐entrapped MGs in self‐assembled structures of cell‐encapsulated MGs. A cell‐tracking study reported an alternative strategy for tissue regeneration involving mesenchymal‐stem‐cell‐internalizable MMGs.^176^ In this study, cellular uptake of iron oxide‐NP‐embedded MGs was used as an MRI tracking mechanism of human fetal mesenchymal stem cells for an in vivo photothrombotic stroke model, without affecting the proliferation and differentiation capabilities of the MG‐bearing stem cells. The study showed the potential of the MGs for clinical application to wound healing.

## CONCLUSIONS

In the first part of this paper, we described the physical engineering principles that are essential for the high‐precision design of MMGs and MNGs, ranging from stimuli‐responsive behavior of hydrogel network, diffusion, and transport phenomena, to colloidal nanomagnetism. In the second part, we related those principles to the biomedical functionality of MMG/MNG systems, summarizing recent progress on these systems and their biomedical and clinically relevant applications. New promising platforms have been reported, and they facilitate on‐demand drug release and show remote controllability under static and alternating magnetic fields. Moreover, extensive research on smart gel synthesis and organic/inorganic functionalization techniques has accelerated the advent of new‐generation hydrogel materials.

Despite the rapid growth of the field, the range of applications does not meet the demands of clinical practices, indicating that the current status of MMG/MNG development is not mature. In order to overcome this barrier and facilitate clinical translation, more predictable and effective systems should be developed based on the precisely tunable properties of nanobio interfaces in vivo. By harnessing more elaborate biofunctionality, including biodegradability and cellular interactions, we could facilitate the introduction of MMGs/MNGs into the clinic. In addition, potential toxic effects of magnetic nanomaterials should be identified to facilitate measures for enhancing their safety. The ultimate objective should be the development of in vivo self‐regulating nanobio‐gel systems that can report local physiological information (via fluorescence or magnetic resonance) and can be magnetically controlled by field manipulators outside the body or 3D complex tissue cultures.

## CONFLICT OF INTERESTS

The authors declare no financial or commercial conflict of interest.

### PEER REVIEW

The peer review history for this article is available at https://publons.com/publon/10.1002/btm2.10190.

## Supporting information


**Appendix S1**: Supporting Information

## Data Availability

Data sharing not applicable to this article as no datasets were generated or analysed during the current study.
