# Supplementary material for: Magnetic microgels and nanogels: Physical mechanisms and biomedical applications
Source: Bioeng Transl Med. 2020 Oct 21;6(1):e10190. doi: 10.1002/btm2.10190 (PMC7823133; doi:10.1002/btm2.10190)
Supplement: Supplementary file 1 — Appendix S1: Supporting Information [file BTM2-6-e10190-s001.pdf]

Supplementary Information

Magnetic Microgels and Nanogels: Physical Mechanisms and Biomedical Applications

Baeckkyoung Sung[1,2,3], Min-Ho Kim[2*], and Leon Abelmann[1,4]

1. *KIST Europe Forschungsgesellschaft mbH, 66123 Saarbrücken, Germany*
2. *Department of Biological Sciences, Kent State University, Kent, OH 44242, USA*
3. *Division of Energy & Environment Technology, University of Science & Technology (UST), 34113 Daejeon, Republic of Korea*
4. *MESA$^+$ Institute for Nanotechnology, University of Twente, 7500 AE Enschede, The Netherlands*

*Correspondence: mkim15@kent.edu

Superparamagnetism

To rotate the magnetization from one direction along the easy axis to the other, an energy barrier should be overcome (Fig. S1A). This barrier decreases with a reduction in the MNP volume. When we reduce the particle size sufficiently (somewhere below 30 nm), the energy barrier becomes comparable to the thermal energy in the MNP (kT), and the magnetic moment spontaneously begins to flip. Consequently, the time-averaged moment of the macrodipole becomes zero and on long timescales, the NP is effectively nonremanent (Fig. S1B). The common term for this effect is superparamagnetism. If we cycle the field very fast, however, the hysteresis loop opens up and hysteretic losses result. This is why we can realize nonremanent MNPs (on timescales of seconds) that can be used for heating (at timescales of milliseconds).

Magnetic domains

For the second method to achieve low remanence, we should consider the internal distribution of atomic spins in the NP. The magnetic energy of a macrodipole is

proportional to the square of the dipole moment. If the MNPs are sufficiently large (usually above 100 nm), the atomic spins have space to form nonuniform patterns and reduce the total dipole moment. In the simplest case, one can imagine that atomic spins in the MNPs can be split into two regions with opposing directions, leading to zero dipole moment. The regions are called domains, and the transition between them is termed domain wall (Fig. S1C).

In the absence of a field, the domains are of equal size and the MNP has zero net magnetic moment, or zero remanence. If we apply a field, the size of the domain with spins aligned in parallel increases at the cost of the antiparallel domain's size. In other words, the domain wall begins to move until all spins are aligned with the field. In optimized materials, this movement can be very fast (on the order of meters per second). In MNPs, the domain walls can be trapped at energy minima, such as imperfections in the crystal or surface irregularities. This trapping leads to hysteresis and energy dissipation.

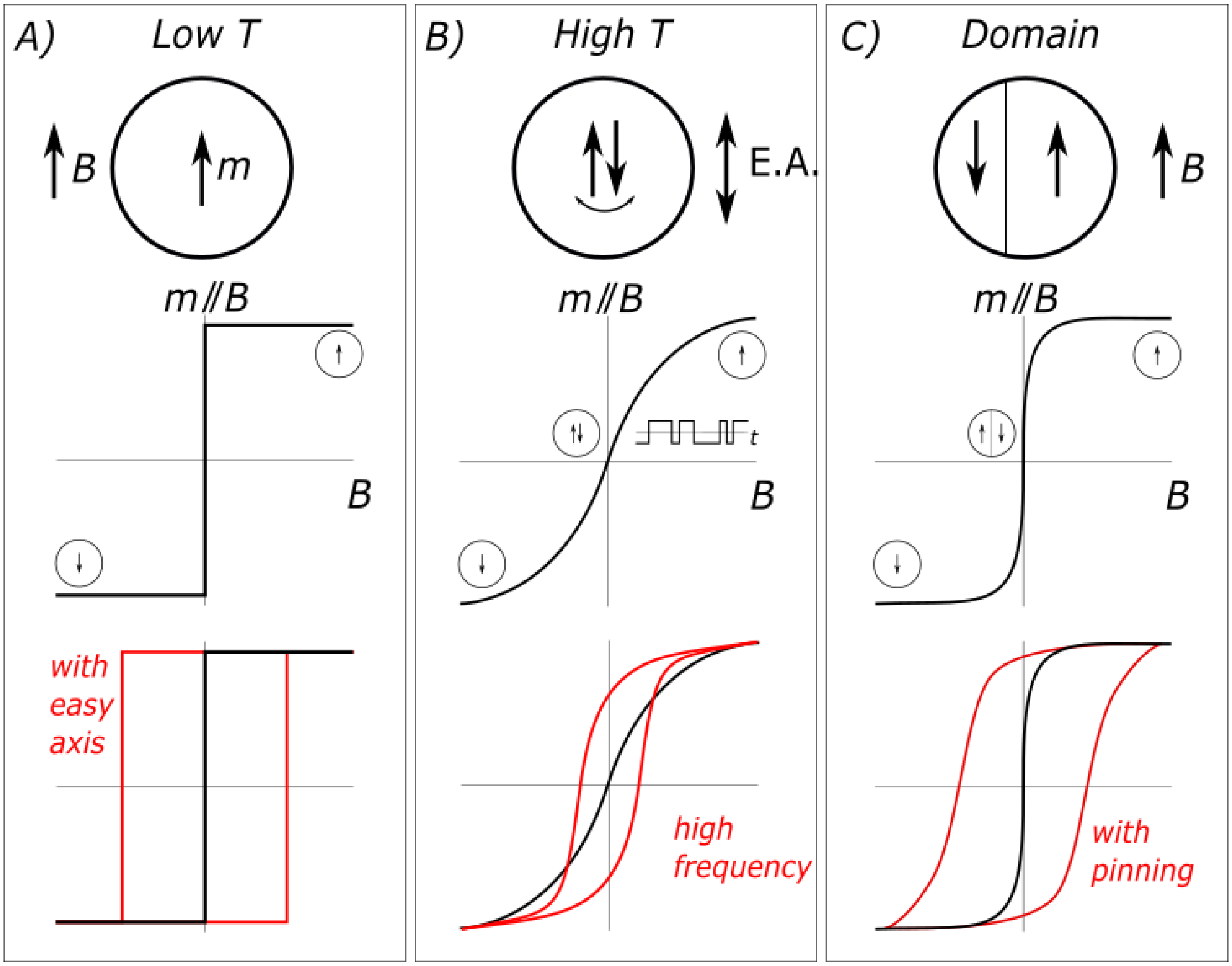

Figure S1: Magnetic reversal of MNPs. (A) When we consider the MNP as a macrodipole, with all atomic spins pointing along the field, the dipole moment simply follows the field, and we obtain a rectangular hysteresis loop (center image). When the MNP has an easy axis of magnetization, there are two stable states at zero field, and the loop opens up (red curve, bottom image) (B) With increasing temperature, the thermal energy in the MNP becomes sufficient to overcome the energy barrier between the two stable states and the magnetic moment begins to “flip” (inset, center figure), leading to zero average remanence. When we sweep the field at a high frequency, the moment no longer has time to flip, and the hysteresis loop opens up again (red curve). (C) In large MNPs, the atomic spins can minimize the energy by forming domains with opposite orientations, leading to zero remanence. If the domain wall between the opposite domains pins on imperfections, the hysteresis loop opens up (red curve).

Table S1: Magnetic microgels and nanogels for bioseparation and biocatalysis

Size	Shape and structure	Polymeric materials	Magnetic materials	Magnetic properties	MNP hybridization method	Magnetic field controls	Applications	Reference
250-450 nm	MNPs-embedded matrix	poly(*N*-isopropylacrylamide)	*In situ* synthesized Fe_3O_4 NPs	N/A	*In situ* co-precipitation of protein gels & MNPs upon addition of Fe^{2+}	N/A	Magnetic bioseparation	Némethy et al., 2008
0.5-3 µm	MNPs-embedded matrix	Poly vinyl phosphonic acid	Pre-synthesized Fe_3O_4 NP (silica-coated)	N/A	Microgel synthesis in the presence of MNPs (ferrofluid)	N/A	Magnetic bioseparation & drug release	Sengel & Sahiner, 2016
26-38 nm	Spherical Fe_2O_3 NP coated with polymer gel	Copolymer of poly(ethylene glycol) & polycarboxylate	Pre-synthesized iron oxide NP (7-10 nm in diameter)	Super-paramagnetic, M_s = 22-25 emu/g	Nanogel shell synthesis in the presence of MNPs (ferrofluid)	Static field application	Magnetic separation of melanoma cells	Sunderland et al., 2006
~10 nm	Spherical MNP coated with polymer gel	Quercetin-templated *N*-vinyl imidazole (crosslinked with Tragacanth Gum)	Pre-synthesized Fe_3O_4/SiO_2 NP	Super-paramagnetic, M_s = 20 emu/g	Nanogel shell synthesis in the presence of MNPs (ferrofluid)	N/A	Magnetic bioseparation & drug release	Hemmati et al., 2016
100-200 nm	Magnetic core (clustered MNPs) & polymeric gel shell	Polyacrylic acid, poly(*N*-isopropylacrylamide), & poly(3-acrylamidopropyl trimethylammonium chloride)	Pre-synthesized Fe_3O_4 NP	Super-paramagnetic, M_s = 43-77 $A{\cdot}m^2/kg$	Microgel shell synthesis in the presence of MNP clusters	Static field application for separation in aqueous solutions	Magnetic bioseparation	Turcu et al., 2015

60-180 nm	Spherical polymer gel where MNPs are embedded	Chitosan	Pre-synthesized Fe_3O_4 NP (~10 nm in diameter)	Super-paramagnetic	Physical adsorption of MNPs in the nanogel matrix	Static field application for separation in aqueous solutions	Magnetic separation of proteins	Gaemy & Naseri, 2012
~570 nm	Spherical MNP coated with thermo-sensitive polymer gel where Au NPs are embedded	Poly[*N*-isopropylacrylamide-*co*-2-(dimethylamino)ethyl methacrylate]	Pre-synthesized Fe_3O_4 NP (~320 nm in diameter)	M_s = 28-36 emu/g	Microgel synthesis in the presence of MNPs (ferrofluid)	Static field application for separation in aqueous solutions	Magnetic purification & sensing with thermo-responsivity	Liu et al., 2015
~10 nm (dried state)	Spherical polymer gel where MNPs are embedded	Oly(4-vinylpyridine)	Pre-synthesized Fe_3O_4 NP	N/A	Microgel synthesis in the presence of MNPs (ferrofluid)	Static field application for separation in aqueous solutions	Magnetic purification with pH-responsivity	Tabani et al., 2015
20-200 nm	Spherical MNP coated with a nanogel layer	Methylacrilic acid & *N,N'*-methylene-*bis*-acrylamide	Pre-synthesized Fe_3O_4 NP (10 nm in diameter)	Super-paramagnetic, M_s = 63 emu/g	Photochemical *in situ* polymerization of nanogel layer on the MNP surface	Static field application for separation in aqueous solutions	Biocatalysis & magnetic separation	Hong et al., 2007
500-700 nm	Spherical polymer gel where MNPs are embedded	Poly(*N*-isopropylacrylamide)-*co*-acrylic acid	*In situ* synthesized Fe_3O_4 NPs	Super-paramagnetic, M_s ~60 emu/g	*In situ* synthesis of MNPs in the microgel templates	Static field application for separation in culture media & whole blood	Magnetic separation of cancer cells	Seyfoori et al., 2019
~32 µm	Porous spherical clay gel where MNPs are embedded	$LiMgNaO_6Si_2$ clay (LAPONITE XLG)	Pre-synthesized iron oxide NPs	N/A	Clay gelation in the presence of MNPs	Static field application for separation in aqueous solutions	Magnetic separation of proteins	Jiao et al., 2018

25-30 µm	Spherical polymer gel where MNPs are embedded	Alginate	Pre-synthesized Fe_2O_3 NP (15-20 nm in diameter)	N/A	Microgel synthesis in the presence of MNPs (ferrofluid)	Static field application for separation in aqueous solutions	Magnetic separation of microgel assemblages	Hu et al., 2017

Table S2: Magnetic microgels and nanogels for *in vivo* imaging and optical sensing

Size	Shape and structure	Polymeric materials	Magnetic materials	Magnetic properties	MNP hybridization method	Magnetic field controls	Applications	Reference
150-200 nm	Spherical Fe_3O_4 NP coated with thermo- & pH-sensitive polymer gel	Poly(*N*-vinylcaprolactam-*co*-itaconic acid)	Pre-synthesized Fe_3O_4 NP (50-60 nm in diameter)	M_s = 0.05-0.2 emu/g	Polymerization of gel layer on the MNP surface	MRI	MRI	Medeiros et al., 2016
~2 mm	Spherical polymer gel where MNPs are embedded	Poly(vinyl alcohol-*co*- *N*-isopropylacrylamide)	Co-precipitated Fe_3O_4 NP	Super-paramagnetic, M_s = 7-11 emu/g	*In situ* co-precipitation of polymer gels & MNPs	Guidance by static fields	Release of diagnostic dye	Zhou et al., 2012
210-230 nm	Spherical polymer gel where MNPs are embedded	Carboxymethyl cellulose	*In situ* synthesized Fe_2O_3 NPs	Super-paramagnetic, M_s ~63 emu/g	*In situ* co-precipitation of protein gels & MNPs	Static field guidance	Drug release & MRI & optical imaging	Bakandritsos, 2011
~110 nm	Spherical polymer gel where MNPs are embedded	Polycarboxybetaine methacrylate	Pre-synthesized Fe_3O_4 NP (9 nm in diameter)	N/A	Nanogel synthesis in the presence of MNPs (ferrofluid)	MRI	Magnetically targeted drug delivery & *in vivo* imaging	Zhang et al., 2011
~200 nm	Spherical polymer gel where MNPs are	Chitosan/acryl/biotin	Pre-synthesized iron oxide	Super-paramagnetic, M_s ~38 emu/g	Microgel shell synthesis in the presence	MRI	pH-responsive MRI contrast	Wang et al., 2015

	embedded		NP (6 nm in diameter)		of MNP clusters		agent	
~300 nm (dried state)	Spherical MNP coated with polymer gel	Poly(ethylene glycol)	Pre-synthesized Fe_3O_4 MNP (~200 nm in diameter)	M_s ~94 emu/g	Nanogel synthesis in the presence of MNPs (ferrofluid)	Static field application for separation in aqueous solutions	Colorimetric detection of glucose for medical diagnosis	Wu et al., 2015
< 200 nm	MNPs are embedded in a spherical matrix of amphiphilic brush copolymers	Alkylamine chains-grafted poly(isobutylene-*alt*-maleic anhydride)	Pre-synthesized Fe_3O_4 nanocrystals (6 nm in diameter)	Super-paramagnetic, M_s = 5-35 emu/g (increases as MNP loading)	Nanogel synthesis in the presence of MNPs (ferrofluid)	MRI	MRI & optical imaging agent	Choo et al., 2011
~200 nm	Polymeric sphere embedding or is decorated by MNPs	Poly(2-vinylpiridine-*co*-divinylbenzene)	Pre-synthesized iron oxide NP (~5 nm in diameter)	Super-paramagnetic, M_s ~60 emu/g	Covalent linking of MNPs during nanogel synthesis	Static field application for separation in aqueous solutions	Colorimetric pH sensing & magnetic manipulation	Riedinger et al., 2011

Table S3: Magnetic microgels and nanogels for general applications

Size	Shape and structure	Polymeric materials	Magnetic materials	Magnetic properties	MNP hybridization method	Magnetic field controls	Applications	Reference
360-840 nm	Spherical polymer gel where MNPs are embedded	Poly(*N*-isopropylacrylamide-*co*-glycidyl methacrylate)	*In situ* synthesized iron oxide NPs (~13 nm in diameter)	N/A	*In situ* synthesis of MNPs in the microgel templates	N/A	N/A	Suzuki & Kawaguchi, 2006
9-30 µm	Spherical polymer gel where MNPs	Poly(acrylamide-*co*-Methylacrylic acid)	*In situ* synthesized Fe_3O_4 NPs	M_s = 2.7 emu/g; coercivity	*In situ* synthesis of MNPs in the microgel templates	N/A	N/A	Tan et al., 2010

	are embedded			= 63.1 Oe				
20-300 µm	Non-spherical polymer gel where MNPs are embedded	Poly(ethylene glycol) & poly(acrylic acid)	*In situ* synthesized Fe_3O_4 NPs (3.5-5.5 nm in size)	M_s < 40 emu/g	*In situ* synthesis of MNPs in the microgel templates	N/A	N/A	Suh et al., 2012
